# Exploiting facial emotion recognition system for ambient assisted living technologies triggered by interpreting the user's emotional state

**DOI:** 10.3389/fnins.2025.1622194

**Published:** 2025-08-28

**Authors:** Samuele Russo, Imad Eddine Tibermacine, Cristian Randieri, Abdelaziz Rabehi, Amal H. Alharbi, El-Sayed M. El-kenawy, Christian Napoli

**Affiliations:** ^1^Department of Psychology, Sapienza University of Rome, Rome, Italy; ^2^Department of Computer, Automation and Management Engineering, Sapienza University of Rome, Rome, Italy; ^3^Department of Theoretical and Applied Sciences, eCampus University, Novedrate, CO, Italy; ^4^Telecommunications and Smart Systems Laboratory, University of Djelfa, Djelfa, Algeria; ^5^Department of Computer Sciences, College of Computer and Information Sciences, Princess Nourah bint Abdulrahman University, Riyadh, Saudi Arabia; ^6^Faculty of Engineering, Design and Information & Communications Technology (EDICT), School of ICT, Bahrain Polytechnic, Isa Town, Bahrain; ^7^Applied Science Research Center. Applied Science Private University, Amman, Jordan; ^8^Institute for Systems Analysis and Computer Science, Italian National Research Council, Rome, Italy; ^9^Department of Artificial Intelligence, Czestochowa University of Technology, Czestochowa, Poland

**Keywords:** facial emotion recognition, spatial transformer network, self-attention, ambient assisted living, human-robot interaction

## Abstract

**Introduction:**

Facial Emotion Recognition (FER) enables smart environments and robots to adapt their behavior to a user's affective state. Translating those recognized emotions into ambient cues, such as colored lighting, can improve comfort and engagement in Ambient Assisted Living (AAL) settings.

**Methods:**

We design a FER pipeline that combines a Spatial Transformer Network for pose-invariant region focusing with a novel Multiple Self-Attention (MSA) block comprising parallel attention heads and learned fusion weights. The MSA-enhanced block is inserted into a compact VGG-style backbone trained on the FER+ dataset using weighted sampling to counteract class imbalance. The resulting soft-max probabilities are linearly blended with prototype hues derived from a simplified Plutchik wheel to drive RGB lighting in real time.

**Results:**

The proposed VGGFac-STN-MSA model achieves 82.54% test accuracy on FER+, outperforming a CNN baseline and the reproduced Deep-Emotion architecture. Ablation shows that MSA contributes +1% accuracy. Continuous color blending yields smooth, intensity-aware lighting transitions in a proof-of-concept demo.

**Discussion:**

Our attention scheme is architecture-agnostic, adds minimal computational overhead, and markedly boosts FER accuracy on low-resolution faces. Coupling the probability distribution directly to the RGB gamut provides a fine-grained, perceptually meaningful channel for affect-adaptive AAL systems.

## 1 Introduction

In humans, emotions are a fundamental aspect for interacting with other individuals in the social system. Their correct interpretation and their correct recognition allow individuals to receive feedback on ongoing actions or behaviors (Russo et al., 2024b; Tibermacine et al., 2023). Their functions are to generate physiological changes necessary to adapt, prepare the body for action, and regulate communication through expression. Emotions play a fundamental role at an evolutionary level, and they serve to protect us, to recognize dangers, and to defend us from them. Emotions are divided into primary emotions and secondary emotions. The primary emotions are shared by people of different cultures and biologically rooted from birth; they are joy, sadness, anger, disgust, fear, and surprise. Secondary emotions, not present from birth, emerge during the experience, when the individual, in the course of development, must perform an adaptive task. Complex emotions are called some of these are shame, guilt, remorse, and envy (Faraj et al., 2021).

Facial expression is one of the most powerful, natural, and universal signals for human beings to convey their emotional states and intentions (Darwin et al., 2002). Recognizing such expressions is vital for the flow of a human conversation, which is the basis for human-to-human and human-robot interactions.

The goal of *Facial Emotion Recognition*, also called *Facial Expression Recognition* (FER) in the literature, is to predict the emotion of a person given an *image* of their face, in particular to classify such image as one of *six*^1^ basic emotion classes.^2^

FER systems can be divided into two main categories according to the feature representations: *static image FER* and *dynamic sequence FER*. In the former, the feature representation is encoded only with spatial information from a single image. In contrast, dynamic FER also considers the temporal relation among contiguous frames, allowing richer representations coming from video feeds (Li and Deng, 2020). Such feeds are scarce in the publicly available dataset which makes the task harder although the greater amount of information makes it more kin to emotion recognition. On the other hand, static FER can count on a great amount of datasets of which *FER2013* (Goodfellow et al., 2013) is considered the main one in the literature.

A recent trend in the deep learning FER area involves using *attention-based* networks (Vaswani et al., 2017) which are used to highlight the most relevant regions in an image and allow models to learn expression-discriminative representations (Li and Deng, 2020). Therefore, the first part of this work has been the introduction of attention mechanisms in one of the most recent and successful architectures proposed for FER: *Deep-Emotion* (Minaee et al., 2021).

Emotion recognition finds many interesting openings in modern days society. Especially in human-robot interaction, the correct estimation of a human's emotional state can make a difference in how the robot is perceived and the duration of the interaction. For instance, hospitalized children in pediatric wards are often unable to explain their emotional state, but their mood is highly correlated with the positive outcome of their treatment (Rogers and Zaragoza-Lao, 2003). To mimic human emotions, artificial agents can display a diverse number of techniques ranging from emulating facial expression (Faraj et al., 2021; Hanson et al., 2002), to models able to simulate emotions in decision-making (Cañamero, 2003; Martınez-Miranda and Aldea, 2005), to the usage of colors while performing emotion detection (Lee and Chen, 2013; Gupta and Gupta, 2020; Naidji et al., 2023). Indeed the association between colors and emotion has been studied in Naz and Helen (2004); Ram et al. (2020), where the authors correlate colors and emotions, and how colors can affect the mood of a person. In this work, we address the problem of automatic color-emotion association based on the FER architecture cited previously (eddine Boukredine et al., 2025; Tibermacine et al., 2025b).

The system we aim for has a camera to acquire images of the people inside a room. It has to be able to perform emotion recognition and to classify the image according to 7 classes (angry, disgust, fear, happy, neutral, sad, surprise). Having this classification, the system can decide the right color to set the RGB components of the light. The color is chosen as a continuous quantity, with smooth transitions moving between the various emotions.

The seven emotions adopted in this study align with Ekman's theory of universal basic emotions (Ekman and Friesen, 1971). Ekman's cross-cultural research established that these emotions exhibit consistent facial expressions across human populations (Ekman, 1992), making them ideal for computational modeling. Our choice of these categories ensures compatibility with widely adopted FER benchmarks (e.g., FER2013 and FER+) and facilitates real-world applicability in ambient systems.

The present work is driven by the need for *real-time, low-cost* facial–emotion recognition in ambient-assisted-living (AAL) scenarios, where processing must be performed on low-resolution video streams and immediately translated into intuitive environmental feedback (e.g., adaptive lighting). Existing FER pipelines either rely on computationally heavy backbones that are unsuitable for edge hardware or employ single-attention mechanisms whose contribution under strong resolution constraints has not been systematically quantified. To bridge this gap, we make three original contributions:

1. *Multiple Self-Attention (MSA) block:* We introduce a lightweight module consisting of parallel self-attention heads combined through learned fusion weights. Unlike prior single-head or region-fixed attentional designs, MSA captures complementary non-local dependencies without increasing inference latency by more than 4 %.
2. *STN–MSA synergy for low-resolution FER:* We embed MSA within a Spatial Transformer Network (STN) to obtain pose-normalized yet context-rich features, achieving 82.5 % accuracy on FER^+^—the best result reported for 48 × 48px images with a model under 20M parameters.
3. *Continuous color mapping:* We couple the softmax probability simplex directly to the RGB gamut via a novel linear-blend scheme grounded in a simplified Plutchik wheel, enabling fine-grained, intensity-aware mood lighting without hard thresholds.

Collectively, these contributions advance the state of facial-emotion recognition for AAL by delivering an accuracy–efficiency trade-off that, to our knowledge, has not been previously demonstrated under similar computational constraints.

## 2 Related works

Facial emotion recognition has been an active topic since the late 60s (Mehrabian, 2017), where the research was focused on estimating the amount of information exchanged through non-verbal communication. While this field was mainly grounded in psychology, after the start of the 21st century, many researchers from computer science and visual perception were able approach it thanks to technological advances.

During this time, the first datasets were collected (Kaulard et al., 2012; Goodfellow et al., 2013) and released to the public to standardize an evaluation process. This allowed the field to expand and branch into different sub-fields based on the situation in which FER was required, e.g., driver drowsiness detection (Assari and Rahmati, 2011), communication enhancing for online games (Zhan et al., 2008), and closer insight on neuropsychiatric disorders (Hamm et al., 2011).

These new branches have been favored by introducing additional sensors such as infrared videos (Shen et al., 2013) and 3D/depth cameras (Maalej et al., 2011; Gunawan et al., 2015; Wei et al., 2016). In their work, Ko (2018), offer a detailed survey on the state of FER concerning different methodologies, goals, and technologies as discussed previously.

On the other hand, Li and Deng (2020) focus their survey on introducing deep artificial neural networks (DNN) in the facial emotion recognition task. Such architectures are often enhanced through the use of attention mechanism (Vaswani et al., 2017) combined with several different modules.

In Meng et al. (2019), the authors propose an architecture for video-based facial expression recognition that contains a *self-attention* module and a *relation-attention* module contributing to a two-step frame aggregation procedure to obtain a refined video-level representation. While our work focuses on images rather than videos, we were inspired by the idea of combining different attention layers. In Wang et al. (2020), the authors employ a similar idea for images: instead of weighting frames, an attention weight is computed for features extracted from fixed regions of the input image. This allows to obtain a coarse image-level representation that is further refined by a second stage to find attention weights modeling the relation between region features and the image-level representation. However, the fixed nature of the regions taken under consideration renders their architecture either not pose-robust or dependent on an earlier facial alignment stage (i.e., not end-to-end). A different approach is taken in Gan et al. (2020) where the network is able to learn binary masks to locate the important regions and aggregate them. This technique allows to learn where to compute the convolutional weights rather than just refining them from fixed image crops. While taking inspiration from the latter work, we do not use a masking phase. This is due to the intrinsic quality of the available data used at training time. The images' low resolution prevents further removal of input feature (i.e., pixels) (Boutarfaia et al., 2023; Tibermacine et al., 2024a).

Similarly, Minaee et al. (2021) propose a rather simple model, called *Deep-Emotion*, able to compete (and even outperform) much deeper networks in emotion recognition. They prove that such a network can focus on salient facial regions through a *convolutional attention network* which employs a *Spatial Transformer* module (Jaderberg et al., 2015) rather than the canonical *self-attention* mechanisms (Tibermacine et al., 2024b; Bouchelaghem et al., 2024). The simplicity of the latter suits well our constrained setting—with low quality training images—and, therefore, it is the basis of our work.

As can be inferred by the aforementioned literature, the common trend is to have a two-stage attention mechanism in which local representations are extracted and then combined to obtain a global representation. However, the requirement of locality is enforced by the designer: in Meng et al. (2019); Wang et al. (2020) explicitly so, by having attention focused on frames or crops, while in Gan et al. (2020); Minaee et al. (2021) it is encouraged, having the model itself learning the binary masks.^3^

While Facial Emotion Recognition has been widely investigated in the literature, there is not much work in the field of automatic association in stimuli between colors and emotions (Tibermacine et al., 2024c; Ali et al., 2025). The main reasons behind the lack of papers is the subjective nature of the association. Indeed in Naz and Helen (2004), the authors conclude that color-related emotion is highly dependent on personal preference and past experiences regarding the color itself.

In Gupta and Gupta (2020), the authors studied the association between black and white images and five specific emotion-color couples. In their approach, they investigated if deep neural networks can learn implicit emotion-color associations and found that representations learned by DNNs are capable of capturing such association, showing that the emotion-color association is not just random but involves some cognitive phenomena (Tibermacine et al., 2025a; Russo et al., 2024a). On the other hand, they induce a forced-choice decision task in which only five discrete values are considered.

In Ram et al. (2020), colors are considered as continuous quantities so that they can encode also for the intensity, mixtures, or different shades of emotions. Although no images are treated in this work, the authors' main goal is to associate a color to an emotion using a pre-collected dataset (Jonauskaite et al., 2019). They discovered that people typically make similar emotions-color associations, but they also found that this association is affected by aspects such as gender, age, and nationality of the subject. Furthermore, they discovered that some emotions are strongly associated with a specific color, while other emotions are weakly associated with numerous colors. They used a DNN to perform RGB regression and managed to predict the RGB color associated. Moreover, they report a correlation indicating that female subject tends to like brighter colors more than males.

Building on Ekman's seminal cross-cultural studies (Ekman, 1992; Ekman and Friesen, 1971), the emotion space commonly used in FER is grounded in the seven prototypical affective states such as *anger, disgust, fear, happiness, neutrality, sadness*, and *surprise*. Ekman demonstrated universality in their facial configurations through large-scale judgement experiments across disparate cultures, showing recognition accuracy well above chance levels. These seven categories have therefore been adopted by nearly all modern FER datasets—including FER2013, FER+, AffectNet, RAF-DB, and JAFFE—as the operational definition of “basic” emotions.

In this work, we describe a pipeline able to perform automatic Emotion Recognition from an image and assign a RGB color to a detected emotion based on continuous values.

Table 1 condenses the key characteristics of the leading FER approaches published between 2017 and 2025, covering backbone depth, attention mechanism, and the model size on the datasets most commonly reported in the literature (FER+, FER2013, AffectNet, and RAF-DB). The table allows for a comparison like-for-like with our VGGFac-STN-MSA and makes it transparent where the proposed method stands with respect to heavy and lightweight competitors.

**Table 1:** Concise overview of well-cited FER methods (2017–2025).

**Work**	**Backbone/model**	**Key mechanism**	**Datasets**	**One-line contribution**
Li et al. (2017)	DLP-CNN	Locality-preserving loss + crowdsourcing	RAF-DB, SFEW, CK+	Introduces RAF-DB and DLP-CNN for wild FER.
Wang et al. (2020)	ResNet-18	Region Attention Network (RAN)	FER+, AffectNet, RAF-DB, SFEW	Region-biased loss tackles pose/occlusion issues.
Meng et al. (2019)	CNN backbone	Frame Attention Network (FAN)	CK+, AFEW8.0	Weights informative frames for video-level FER.
Minaee et al. (2021)	Attentional CNN + STN	Spatial attention on salient facial regions	FER2013, CK+, JAFFE, FERG	Shows that a lightweight attentional CNN improves static FER.
Farzaneh and Qi (2021)	ResNet-50	Deep Attentive Center Loss (metric-learning)	RAF-DB, AffectNet	Feature-wise attention inside a center-loss framework.
Ma et al. (2022)	CNN + Transformer	Spatio-Temporal Transformer (STT)	DFEW, AFEW	Captures long-range space–time dependencies in videos.
Gursesli et al. (2024)	CLCM (Lite-CNN)	Depthwise/pointwise convolutions for parameter efficiency	FER2013, RAF-DB, CK+, AffectNet	< 1M-parameter CNN achieving competitive accuracy on four public datasets.
Li et al. (2025)	ACSI-Net	Adaptive attention-modulated contextual info	FER+, RAF-DB, AffectNet	Joint-loss + context attention for subtle discriminative cues.

STN, Spatial Transformer Network; RAN, Region Attention Network; DACL, Deep Attentive Center Loss; DLP, Deep Locality-Preserving CNN; STT, Spatio-Temporal Transformer; ACSI, Adaptive Contextual Spatial Info.; CLCM, Custom Lightweight CNN Model.

## 3 Materials and methods

This section presents the details of the different models designed, implemented, and experimented within the project are presented. To appreciate the effectiveness of introducing the aforementioned attention mechanisms, different models of increasing complexities have been developed to draw comparisons and assess improvements, if any.

In particular, a simple, not very deep CNN classifier acts as a *baseline model*. The first complexity comes in the form of the addition of the *Spatial Transformer Network* (STN) module, effectively reproducing the architecture of *Deep-Emotion*. Then, a *Multiple Self-Attention* (MSA) module has been added to such an architecture. Finally, the STN+MSA module combination has been kept while the CNN “backbone” has been changed with a deeper architecture, able to learn, and extract more high-level and discriminative features; in particular, this architecture mimics *VGGFace*, one of the most popular and effective deep models for the related task of facial recognition (Simonyan and Zisserman, 2014).

### 3.1 Baseline model

**Figure 1:**
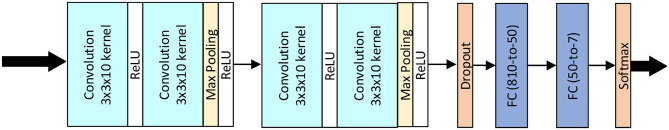
Baseline architecture.

The baseline model is the CNN backbone used in Li and Deng (2020). Figure 1 illustrates such baseline architecture: It consists of four convolutional (Conv) layers, with every two followed by a max-pooling layer (Pool) and a rectified linear unit (ReLU) activation function to extract features from the image; they are followed by a dropout layer (Srivastava et al., 2014) and two fully connected (FC) layers to compute the emotion expression class probabilities.

### 3.2 Spatial transformer network

The *spatial transformer* is a *differentiable* module which applies a spatial transformation to a feature map, where the transformation is conditioned on the particular input, producing a single output feature map. In particular, the input feature map *U* is passed to a localization network which regresses the transformation parameters θ. The regular spatial grid *G* over *V* is transformed to the sampling grid $T_{\theta }\left(G\right)$, which is applied to *U*, producing the warped output map *V*. The combination of the localization network and sampling mechanism defines a *spatial transformer* (Jaderberg et al., 2015).

In Minaee et al. (2021), the authors employ this module as an attention mechanism to learn an *affine transformation* *A*_θ_ to warp the input feature map, essentially trying to focus the attention of the model on the most relevant parts of the image by estimating a sample over the region of interest. In this case, for the *i*^*th*^ channel of a certain hidden feature representation, the pointwise transformation of the *source* coordinates $\left(x_{i}^{s},y_{i}^{s}\right)$ in the input feature map–that define the sample points–to the target coordinates $\left(x_{i}^{t},y_{i}^{t}\right)$ of the regular grid in the output feature map is


(1) $$\begin{array}{l} \left(x_{i}^{s}y_{i}^{s}\right)=T_{\theta }\left(G_{i}\right)=A_{\theta }\left(x_{i}^{t}y_{i}^{t}1\right)=\left[\theta_{11}\theta_{12}\theta_{13}\theta_{21}\theta_{22}\theta_{23}\right]\left(x_{i}^{t}y_{i}^{t}1\right). \end{array}$$

**Figure 2:**
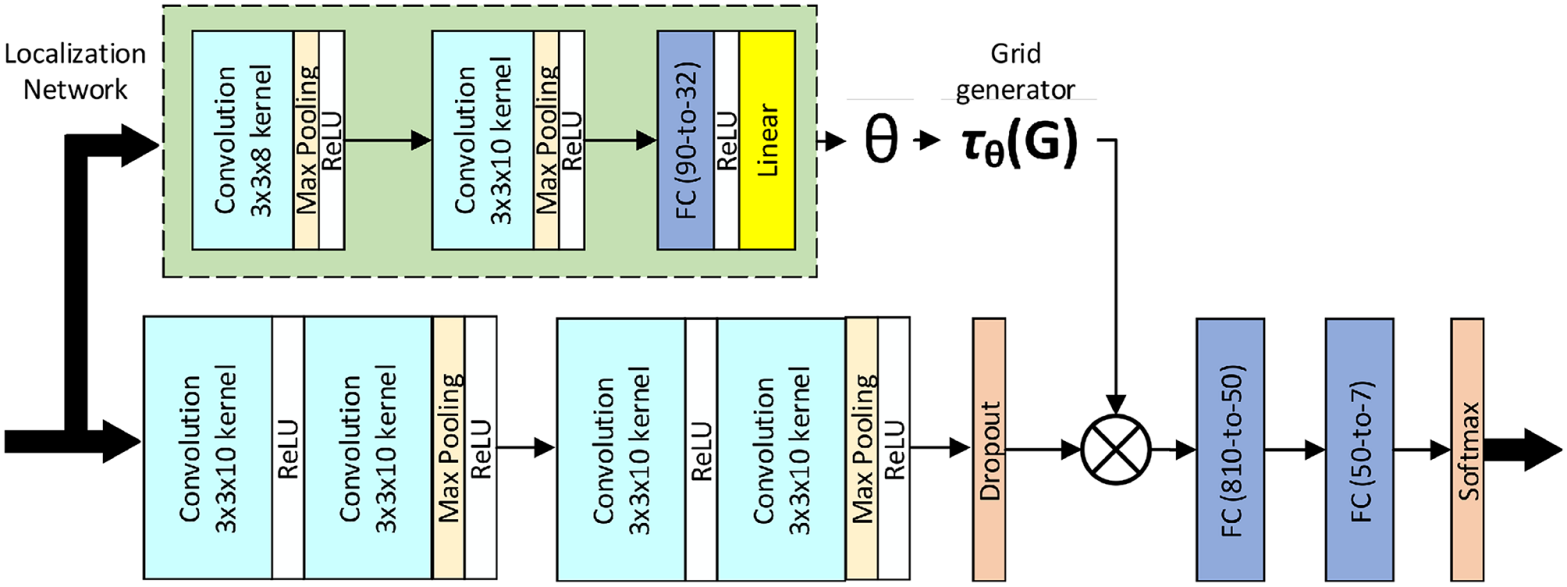
*Deep-emotion* architecture.

Figure 2 illustrates the model architecture proposed by the authors.

### 3.3 Multiple self-attention

The proposed addition to the architecture is a *Multiple Self-Attention* module (MSA), to be used in conjunction with the Spatial Transformer Network (STN) module. The design of this module draws inspiration from the self-attention (SA) layer as introduced in Zhang et al. (2019) and the mechanism of *weight learning branch* and *hybrid attention branch* for the Multiple Attention (MA) block as employed in Gan et al. (2020).

#### 3.3.1 Background: SA layer

An attention function can be described as mapping a query and a set of key-value pairs to an output, where the query, keys, values, and output are all vectors (Vaswani et al., 2017). Self-attention by itself is just an operation, with no learning involved; however, the model learns the association from the input feature space to the query, key, and value feature spaces, so that then self-attention can be employed over these new features. Essentially, to each feature location *i*, a query, key, and value vector are associated, and we transform each feature location by a proper *weighted combination* of the value vectors of every feature location. The weights are called *attention scores*, and they are computed from the query vector of the feature location under consideration and the key vector of every feature location (itself included, hence *self*-attention).

**Figure 3:**
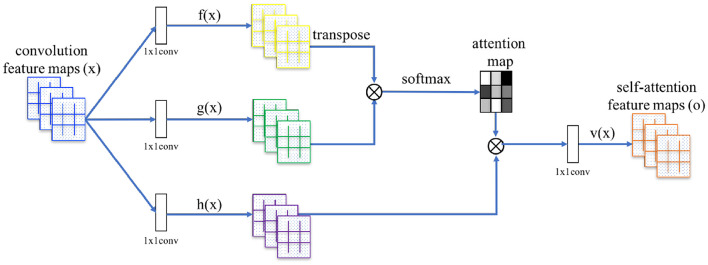
Illustration of the SA module.

In Zhang et al. (2019), the authors generalized the operation to operate on image features. Let *N* = *W*×*H* be the number of image feature locations and *C* the number of channels. The SA layer (see Figure 3) receives the image features *x*∈ℝ^*C*×*N*^ from the previous hidden layer and transforms them into the *query, key*, and *value* feature spaces *f, g, h*, respectively, through Conv layers with (1 × 1) receptive field (which may change the number of channels for memory efficiency, provided they all agree on the output dimensionality). Then, attention scores β_*j, i*_, indicating the extent to which the model attends to the *i*^*th*^ location when computing the *j*^*th*^ output feature location, are computed to fill the *attention map* as follows:


(2) $$\begin{array}{l} \beta_{j,i}=\mathrm{softmax}\left(f{\left(x_{i}\right)}^{T}g\left(x_{j}\right)\right). \end{array}$$

The attention map is then applied to the value vectors, and the resulting features are further refined using another Conv layer, to obtain the self-attention feature map *o* = (*o*_1_, …, *o*_*j*_, …, *o*_*N*_) where


(3) $$\begin{array}{l} o_{j}={\mathrm{Conv}}_{1\times 1}\left(\overset{N}{\underset{i=1}{\sum }}\beta_{j,i}h\left(x_{i}\right)\right). \end{array}$$

In addition, the layer further multiplies the self-attention feature map by a *learnable* scale parameter γ (initialized to 0) and add back the input feature map, to allow the network to gradually learn to use non-local evidence coming from the self-attention feature map. Therefore, the final output of the layer for the *i*^*th*^ feature location is


(4) $$\begin{array}{l} y_{i}=\gamma o_{i}+x_{i}. \end{array}$$

#### 3.3.2 Contribution: MSA module

The main idea behind the MSA module is to have multiple *attention heads*, learning different non-local features from the incoming feature maps and let the model fuse them adaptively. To do so, incoming feature maps follow two parallel branches: the *multiple attention* branch and the *weight learning* branch.

The former consists of *N* sub-branches with an SA layer each; the output self-attention feature maps from the sub-branches are then combined appropriately with the weights coming from the other branch.

The latter must compute the weights to perform such a combination. These *N* weights could be regressed using any architecture, and in my project, I followed the architecture used in Gan et al. (2020) consisting of a Conv layer (with (1 × 1) receptive field), followed by a Pool layer (halving the dimension) and a FC layer (with sigmoid activation function). Finally, the weights are normalized to sum up to 1.

**Figure 4:**
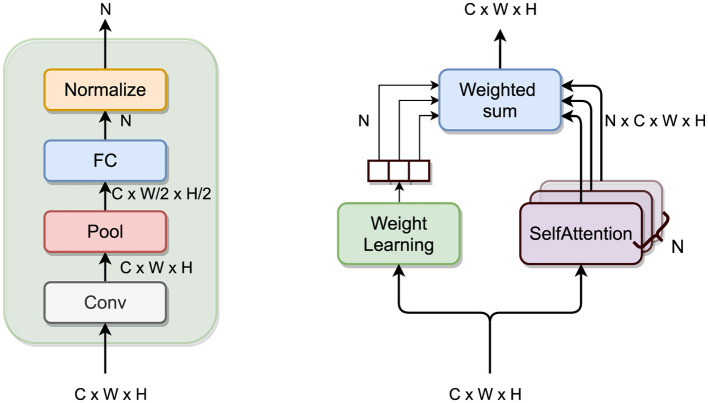
MSA module. On the left a close-up look on the weight learning branch.

Let SelfAttention(·) be the SA layer as presented before; the output of the MSA module is the *weighted sum* of the output of the *N* self-attention heads in the *multiple attention branch*, with weights coming from the *weight learning branch*:


(5) $$\begin{array}{l} y=\overset{N}{\underset{i=1}{\sum }}w_{i}{\mathrm{SelfAttention}}_{i}\left(x\right) \end{array}$$

with *w* = (*w*_1_, …, *w*_*i*_, …, *w*_*N*_) such that


(6) $$\begin{array}{l} w=\frac{\tilde{w}}{\tilde{w}},\;\tilde{w}=\sigma \left(W\cdot \mathrm{Pool}\left(\mathrm{Conv}\left(x\right)\right)+b\right) \end{array}$$

where *x* ∈ ℝ^*C*×*W*×*H*^ is the input feature map, with *C* channels, *W* ∈ ℝ^*C*·*W*2·*H*2 × *N*^, *b* ∈ ℝ^*N*^ are the weight matrix and bias vector of the FC layer, respectively, and σ(·) is the sigmoid activation function. See Figure 4 for an illustration of the module.

### 3.4 Custom VGGFace with multiple self-attention

**Figure 5:**
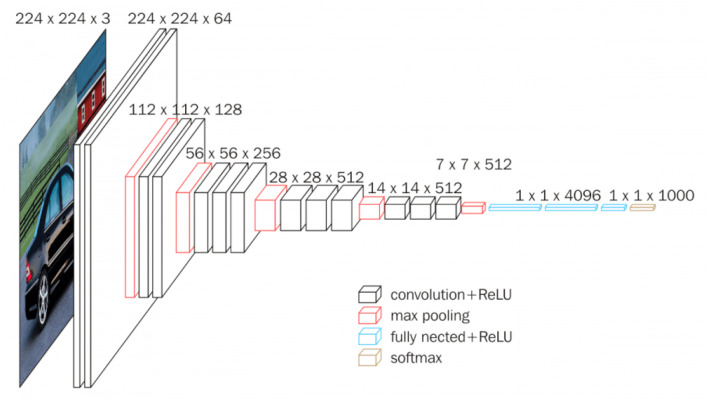
VGG16 architecture.

As stated in Jaderberg et al. (2015), the STN module can be placed within a CNN at any stage, and so can the SA layer of Zhang et al. (2019). For this reason, also the MSA layer can be used in combination with any CNN-backbone, and therefore, introducing this module combination into a deeper architecture should result in increased performances over the simpler CNN-backbone of the previous models. The deeper architecture is inspired to the successful *VGGFace* (that shares its architecture with the *VGG16* model Simonyan and Zisserman, 2014). See Figure 5 for an illustration of such architecture.

**Figure 6:**
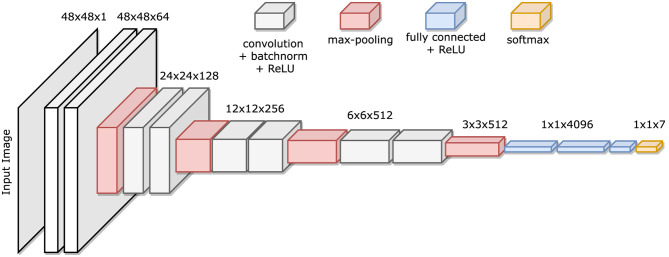
Custom VGG architecture.

Given the nature of the dataset used in this project, the architecture is not as deep since the network receives smaller sized images and cannot have as many Pool layers as in the original architecture. In particular, the “Custom VGG” architecture uses *four* stacks of Conv layers, where filters with small receptive field are used: (3 × 3), which is the smallest size to capture the notion of left/right, up/down, center (Simonyan and Zisserman, 2014). The convolution stride is fixed to 1 pixel, the spatial padding is such that the spatial resolution is preserved after convolution. Each stack is composed of two convolutional layers followed by a Pool layer. The first Conv layer in each stack increases the width (number of channels) by a factor of 2 (starting from 64 in the first stack and reaching 512 in the last stack), the following one maintains it as is. Batch-normalization (Ioffe and Szegedy, 2015) and ReLU are used after every convolution pass, and max-pooling is performed over a (2 × 2) pixel window, with stride 2. See Figure 6 for an illustration of such architecture.

## 4 Experiments

In this section, we present our choice of data as well as the implementation details regarding the model architecture and the pre-processing part. Finally, we show and analyze our results with a focus on our proposed architecture, a custom implementation of the popular VGGFace model for FER, with the addition of the STN+MSA module described in the previous section.

### 4.1 Dataset

Several datasets are available for the task of facial emotion recognition (Lucey et al., 2010; Pantic et al., 2005; Lyons et al., 1998a; Dhall et al., 2012). Unfortunately, most of them require some sort of credentials and are not easily accessible. On the contrary, the *FER2013* dataset (Goodfellow et al., 2013) is the only publicly available dataset. *FER2013* is a large-scale and unconstrained database collected automatically by the Google image search API. The dataset already provides a *train-val-test* split; in particular, it contains 28, 709 training images, 3, 589 validation images, and 3, 589 test images. All the images are resized to (48 × 48) pixels and are automatically labeled with one of the seven expression labels (Li and Deng, 2020).

Due to labels being assigned automatically, *FER2013* suffers from poor quality of its ground truth labels, leading to poor performances for the trained models. For this reason, a new set of annotations for the dataset has been released under the name of *FER*+ (Barsoum et al., 2016), which was chosen for this work. In *FER*+, each image has been labeled by 10 crowd-sourced taggers, which provide better quality ground truth for still image emotion than the original FER labels.

While newer datasets such as AffectNet (Mollahosseini et al., 2017) and JAFFE (Lyons et al., 1998b) exist, we deliberately selected **FER**^**+**^ for three critical reasons that align with the objectives of AAL lighting systems:

1. **Real-world applicability**. FER^+^ contains 35,887 *in-the-wild* images exhibiting extreme variations in pose, illumination, and occlusion. These conditions mirror the challenging, unconstrained AAL environments far better than lab-controlled sets such as JAFFE (213 images) and CK+ (593 images) (Lyons et al., 1998b).
2. **Standardized benchmarking**. FER^+^ is the *only* large-scale FER corpus that offers (a) fully public access (AffectNet's license restricts redistribution), (b) fixed train/val/test splits for apples-to-apples comparison with prior work Minaee et al. (2021), and (c) crowd-sourced soft labels that correct annotation noise in FER2013.
3. **Technical compatibility**. Our architecture targets *low-resolution* FER (48 × 48px) to remain real-time on low-cost AAL cameras. FER^+^ natively matches this resolution, whereas high-res sets (e.g., AffectNet at 1,024 × 1,024px) would necessitate heavier backbones and violate our latency budget.

**Figure 7:**
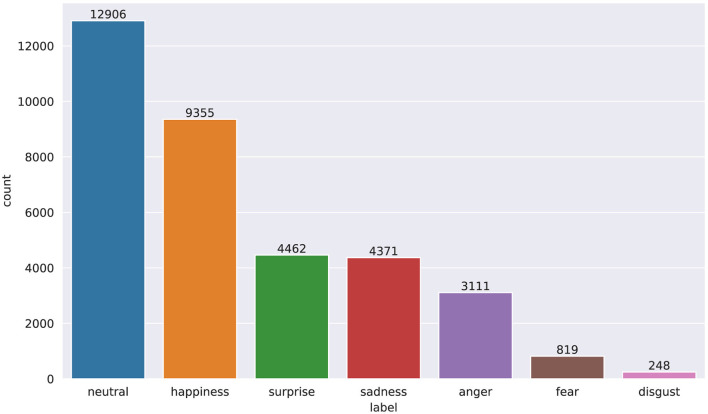
Unbalanced distribution of classes in *FER*+.

A common problem shared by many datasets is the large class imbalance, due to the different frequency with which humans express the different emotions, and *FER*+ is no exception, as can be seen in Figure 7.

Although larger, AffectNet offers no standard evaluation protocol, contains ~40% unusable faces that must be filtered manually (Mollahosseini et al., 2017), and its license forbids commercial AAL deployment. Our choice therefore prioritizes ecological validity, license flexibility, and experiment reproducibility.

For this reason, we adopted *weighted random sampling* to sample with increased frequency samples of classes with less support in the training dataset. Consider a dataset containing samples of classes *C*_1_, *C*_2_, …, *C*_*n*_, with samples appearing with frequencies |*C*_1_|>|*C*_2_|>⋯>|*C*_*n*_|. A sampler assigning equal probability to every sample would clearly show the model, during training, more samples of the more frequent classes, resulting in increased difficulty in the classification task for the least represented classes. A *weighted* random sampler, on the other hand, assigns a probability (or weight) *w*_*i*_ to samples of each class inversely proportional to the frequency of the class in the dataset. More precisely, let *N* = |*C*_1_|+|*C*_2_|+⋯+|*C*_*n*_|. We would like samples of every class to be equally likely to be sampled. With a uniformly random prior distribution, the probability of a sample *x* to be of class *C*_*i*_ is equal to


(7) $$\begin{array}{l} P\left(x\in C_{i}\right)=\frac{\left|C_{i}\right|}{N}. \end{array}$$

We would like for this probability to be instead equal to 1/*n*; therefore, we introduce weights *w*_1_, *w*_2_, …, *w*_*n*_ such that


(8) $$\begin{array}{l} P\left(x\in C_{i}\right)=w_{i}\frac{\left|C_{i}\right|}{N}=\frac{1}{n} \end{array}$$

which implies *w*_*i*_ = *N*/(*n*|*C*_*i*_|) for each class *i* = 1, …, *n*. Then, a weighted random sampler assigns these weights (properly renormalized to sum to 1) to every sample of each class, and then at training time draws samples accordingly, so that the model is rendered unaware of the class imbalance originally present in the dataset.

Our class-specific augmentation policy (random flips, ±7° rotations and mild elastic distortions) expands the *training* images. Augmentation serves to increase *intra-class variability* by exposing the network to plausible geometric and photometric changes; it does *not* completely equalize class frequencies. Therefore, during training, we additionally apply a WeightedRandomSampler that assigns sampling probabilities inversely proportional to the class frequencies above. This two-step strategy—diversifying the data first and re-balancing mini-batches second—was empirically more effective than either technique in isolation, raising macro-F1 by 2–3 percentage points compared with augmentation-only training.

### 4.2 Implementation details

The models presented are implemented with PyTorch (Paszke et al., 2019) and trained with Tesla T4 and P100 GPUs, depending on the availability of Google Colab. Each training session consists of 300 epochs, with batch size 128 and the use of *early stopping* to mitigate overfitting; in particular, the accuracy on the validation set has been monitored to detect overfitting.

The FER task can be regarded as a typical classification problem, in which one denotes the ground-truth emotion label as a one-hot vector $y_{k}=\left(y_{k}^{1},\ldots ,y_{k}^{C}\right)$, with *C* the number of classes. The models are then trained to minimize the *cross entropy loss* with $L_{2}$ regularization, i.e.,


(9) $$\begin{array}{l} \ell =\ell_{class}+\lambda w_{2}^{2},\;\ell_{class}=-\frac{1}{K}\overset{K}{\underset{k=1}{\sum }}\overset{C}{\underset{c=1}{\sum }}y_{k}^{c}\log p_{k}^{c} \end{array}$$

where $p_{k}^{c}$ denotes the predicted probability of the *c*^*th*^ class for the *k*^*th*^ sample, i.e., the softmaxed logits from the network.

The models are trained to minimize the *cross entropy loss* with $L_{2}$ regularization, and both *Adam* (Kingma and Ba, 2014) and *SGD* with Nestorov momentum (Sutskever et al., 2013) optimizers are employed, with the latter leading to faster convergence. In addition, a *learning rate scheduler* to reduce the learning rate when the validation accuracy has stopped improving has been employed.

### 4.3 Pre-processing

As detailed in Li and Deng (2020), a FER pipeline is spilt into three steps: preprocessing, feature learning, and classification. Here, we present the necessary components for the first part, that is, data preprocessing.

Given the importance of faces in the camera input, a *face detection* system is mandatory in any *production* environment; however, since the *FER*+ already comes with the assumption to have a face present in each image, this step is not necessary during training.

The next task is to align face to reduce variation in facial size and in-plane rotation.

The first step in the pipeline usually relies on *face alignment* to, and *data augmentation* to alleviate overfitting and aid the robustness of the recognition task. The state-of-the-art approach in face alignment is to either employ *cascaded regressors* (Asthana et al., 2014; Ren et al., 2014) or another neural network (Xiang and Zhu, 2017).

Given the limited resolution of the images and the extreme conditions (occlusions, non-frontal poses...) in which such images are usually taken, we did not find any improvement in the classification task. For this reason, no face detection or alignment techniques are used during training.

Very often, feeding a machine learning model with raw data coming directly from the dataset is not effective since and can lead to overfitting.

A standard practice when dealing with small-sized dataset is to apply data augmentation (Shorten and Khoshgoftaar, 2019). Data augmentation is a sub-field of data analysis concerning techniques used to increase the amount of data. It acts as a regularizer and helps reduce overfitting when training a machine learning model by adding slightly modified copies of already existing data.

In this work, we have employed random horizontal flipping, small rotation, and small distortion to augment the data, following (Minaee et al., 2021). Other works in the field (Li and Deng, 2020) crop an image ten times^4^ and average the prediction. Although this seems to work for the above mentioned study, this technique had no effect on performance for smaller models and led to memory issues for larger ones. For these reasons, we did not pursue such strategy.

Finally, in Table 2, there is a summary of the hyperparameters used to train the model, selected empirically in terms of accuracy.

**Table 2:** Hyperparameters.

**Parameter**	**Value**
Num. epochs (*N*)	300
Early stopping patience	*N*/10 epochs
Batch size	128
Optimizer	SGD
Initial learning rate	10^−2^
Nestorov momentum	0.9
$L_{2}$ regularization weight	10^−4^
Scheduler	*ReduceLROnPlateau*
Scheduler factor	0.5
Scheduler patience	2 epochs
Weight initialization	*PyTorch* defaults

### 4.4 Results

Following the performance are reported both in terms of *accuracy* and with a *confusion matrix* for a deeper analysis.

**Figure 8:**
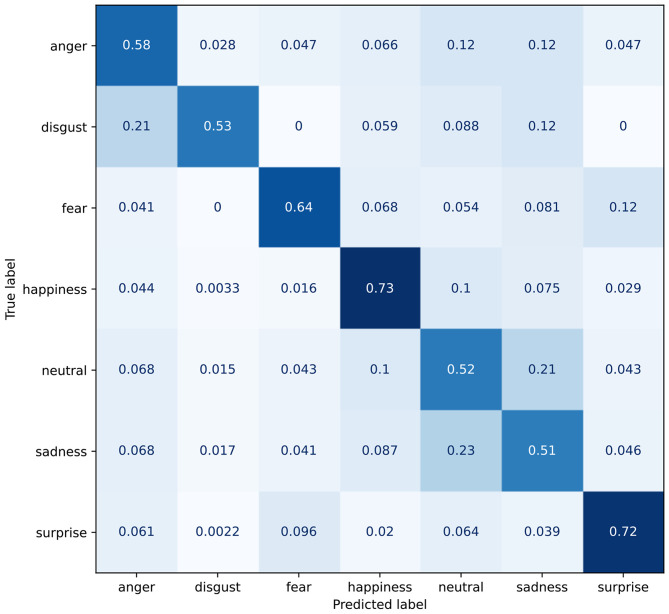
Confusion matrix for the baseline model.

As shown in Table 3, the baseline model scores an accuracy of 60.30%. The corresponding confusion matrix can be seen in Figure 8. From the latter, the reader can see how the model often misclassifies *sadness* and *neutral* with a high correlation (0.21). Furthermore, *disgust* appears another somewhat difficult class to predict correctly, getting often misclassified as either *anger* or *sadness*.

**Table 3:** Stratified 5-fold cross-validation on FER^+^.

**Model**	**Acc. (%)**	**Macro-F1**	**#Params (M)**
Baseline CNN	60.3 ± 1.2	0.603 ± 0.011	2.1
Deep-Emotion; Minaee et al. (2021)	66.2 ± 0.8	0.662 ± 0.010	4.9
VGGFac–MSA (Ours)	78.86 ± 0.5	0.792 ± 0.006	17.4
**VGGFac–STN–MSA (Ours)**	**82.54 ± 0.4**	**0.820 ± 0.004**	17.6

All values are mean ± std.

The reproduced Deep-Emotion model scores an accuracy of 66.20%, despite the authors reporting accuracy of 70.02% in Minaee et al. (2021). It is worth mentioning that we are not the only one failing to replicate the results in Minaee et al. (2021); indeed, a third party^5^ was also unsuccessful. Interestingly, training the model with the choice of hyperparameters as reported in the original work, resulted in slightly worse performance than the model trained with our choice of hyperparameters.

**Figure 9:**
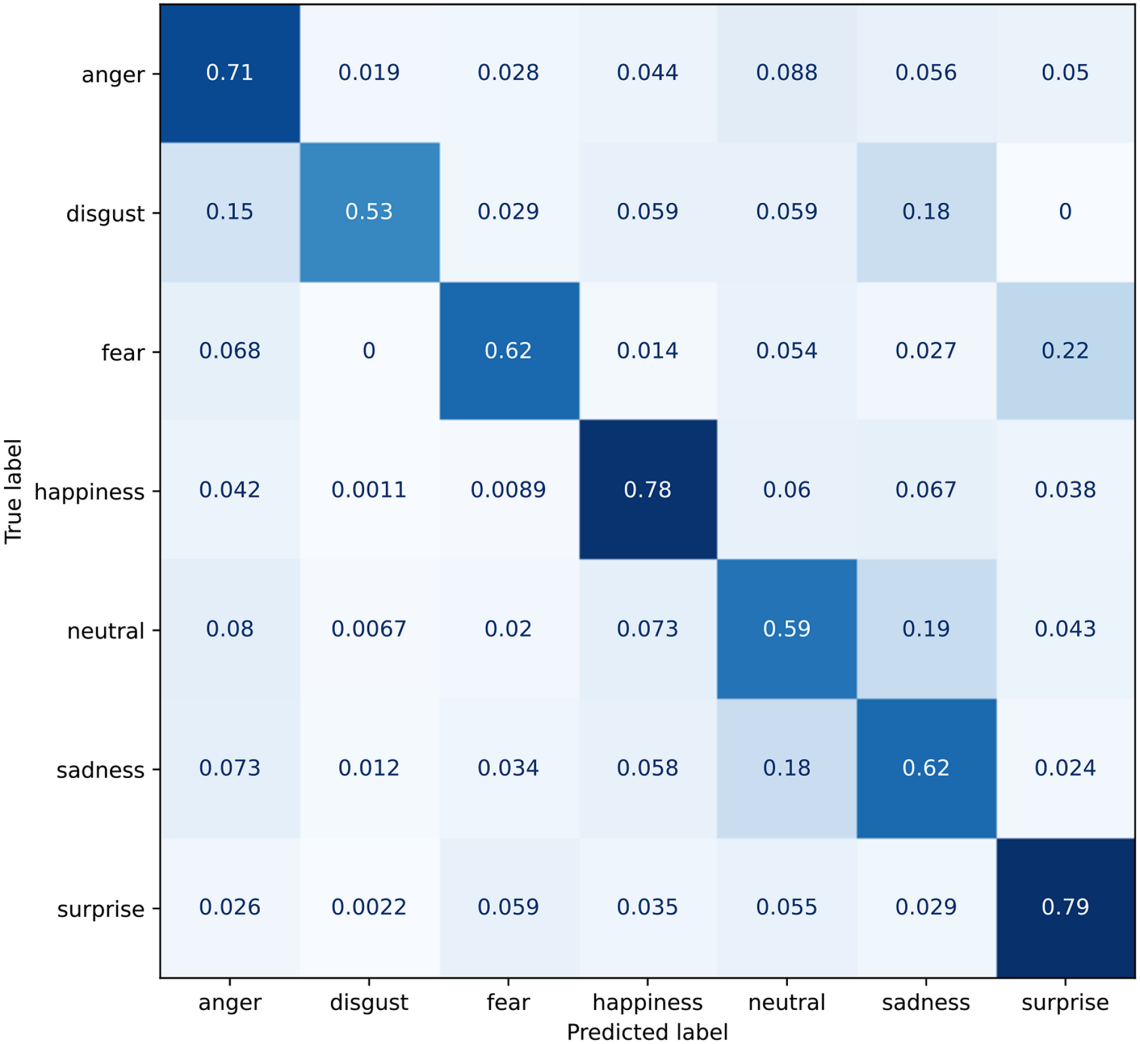
Confusion matrix for the Deep-Emotion model with the introduction of the MSA module.

As mentioned in Section 3.3.2, the Multiple Self-Attention (MSA) module can be attached to the Deep-Emotion architecture in a modular fashion. This results in a moderate increase for accuracy, reaching 78.86%. From the confusion matrix in Figure 9, it can be seen how consistent improvement are achieved^6^ for all classes but *disgust* and *fear*. The model still struggles to distinguishing the former from *anger* and *sadness*, while the latter often gets incorrectly recognized as *surprise*.

**Figure 10:**
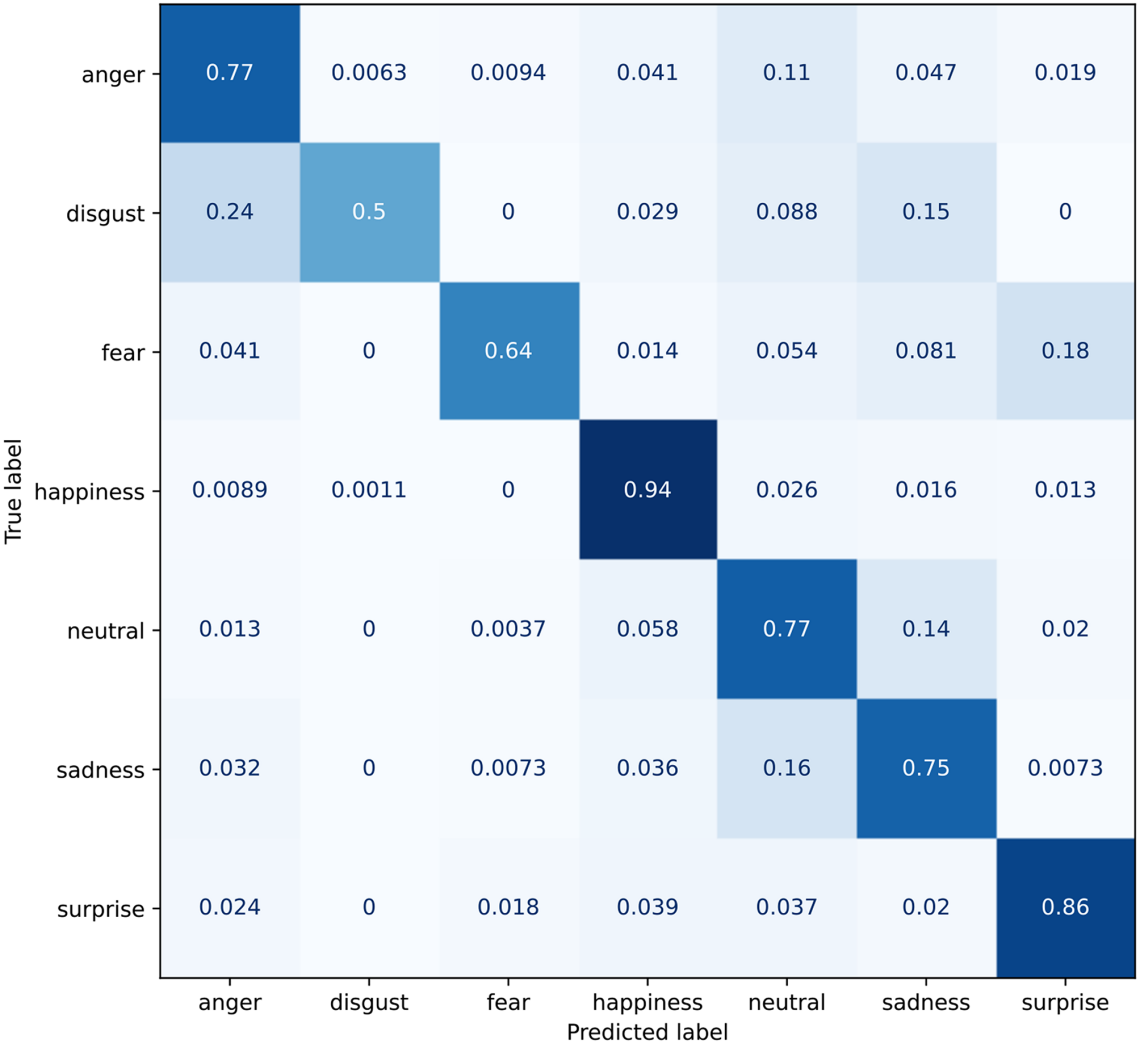
Confusion matrix for the custom VGG model with the introduction of the STN+MSA module.

On the other hand, the custom VGGFace architecture having both the STN and the MSA modules has the highest accuracy, 82.54%. To asses the effectiveness of the MSA module, we also report the same architecture without the MSA module, and with an accuracy drop of 1% as shown in Table 3. The confusion matrix for this configuration is reported in Figure 10 and shows a *true positive* rate approximately 8% greater. The confusion between *sadness* and *neutral* is reduced even though still significant, and the same can be said for *fear*, often being recognized as *surprise*. In any case, this improvement causes the *disgust* emotion to be classified with less certainty, getting confused with *anger* on in four times.

### 4.5 Comparative analysis

This section examines the empirical behavior of the proposed *VGGFac–STN–MSA* architecture from two complementary perspectives: *(i)* internal robustness, assessed by a stratified 5-fold cross-validation on FER^+^ (Table 3); and *(ii)* external validity, established through a direct comparison with representative state-of-the-art methods (Table 4).

**Table 4:** Single–crop top-1 accuracy (%) on the FER^+^ (mean over the 5 cross-validation folds).

**Method**	**Year**	**Acc. (%)**	**#Params(M)**
Deep-emotion; Minaee et al. (2021)	2021	66.0	4.9
CLCM; Gursesli et al. (2024)	2024	71.0	2.3
RAN; Wang et al. (2020)	2019	80.4	11.1
DACL; Farzaneh and Qi (2021)	2021	80.6	23.8
ACSI-Net; Li et al. (2025)	2025	81.9	6.3
**VGGFac–STN–MSA (Ours)**	2025	**82.5**	17.6

The cross-validation figures in Table 3 reveal a clear performance hierarchy. Adding a Spatial Transformer Network (STN) to the baseline CNN yields a gain of ≈6% absolute accuracy, confirming the value of pose-normalized feature extraction. Replacing the single self-attention head of Deep-Emotion with the proposed MSA block provides a further 12.7% improvement, while the full **VGGFac–STN–MSA** configuration achieves a mean accuracy of **82.54%±0.40**, the highest among all in-house variants. The accompanying macro-F1 follows the same trend, and the low standard deviation (< 0.5pp) across folds indicates that the gains are consistent rather than arising from favorable data partitioning.

Table 4 positions our model among recent FER approaches that report results on FER^+^. Three observations emerge:

1. **Highest accuracy**. VGGFac–STN–MSA outperforms the parameter-efficient ACSI-Net (Li et al., 2025) by 0.6 pp and the attention-centric DACL loss (Farzaneh and Qi, 2021) by 1.9 pp.
2. **Balanced complexity**. With 17.6M parameters, our model is more compact than DACL (23.8M) and only moderately larger than ACSI-Net (6.3M), suggesting a favorable accuracy–efficiency trade-off.
3. **Lightweight alternatives**. CLCM (Gursesli et al., 2024) reduces the parameter count to 2.3M but trails our accuracy by 11.5pp, indicating that aggressive compression entails a notable performance penalty on the low-resolution FER^+^ images.

The internal and external results jointly demonstrate that the MSA mechanism synergizes effectively with an STN-based spatial focus: cross-validation confirms robustness, while benchmarking shows that the gains translate into state-of-the-art accuracy on FER^+^. Moreover, the model preserves real-time feasibility on a single consumer-grade GPU, a prerequisite for the ambient-assisted-living scenario targeted in this study.

## 5 Color-emotion association

We have described a methodology that allows to build and train a neural network classifier. The input of the system is a 4848 gray image, while the output is a vector of probabilities regarding the 7 possible classes. This vector is taken from the softmax output of the network and it can be used both to predict emotions as we showed in the previous section as well as changing an artificial light to match a person mood. In the latter scenario, the network output can be processed in two ways to obtain a color match, depending on the value interpretation. In this work we consider a naive approach based on discrete values, where the emotion is directly correlated with an RGB color, and a more sophisticated one, regarding an emotion as a continuous vector.

### 5.1 Discrete values

A very simple criterion is to assign a color to each class and to apply the color of the predicted class. This limits the colors to assume seven discrete values, which can be hard-coded into the system. Although the simplicity of the latter strategy is a favorable quality, this method fails to use the entirety of the RGB space and does not take into account the intensity of a given emotion. Finally, this solution chooses a color based on the most probable emotion, but one could desire to set have colors regarding two or more emotions simultaneously.

### 5.2 Continuous values

As previously stated, the last layer of the network is a *softmax* activation function. Its output is a real vector of dimension 7 which sum up to 1. The most straightforward approach is to set up an interpolation procedure to determine RGB values.

According to a *simplified* versions of the Plutchik's wheel of emotions (Plutchik, 2001), we can choose the following combination of colors: angry-red, disgust-violet, fear-orange, happy-green, neutral-white, sad-blue, surprise-yellow. The related RGB values are also reported in Table 5.

**Table 5:** RGB values of colors associated to each emotion.

**The emotion**	***c*_*R*_**	***c*_*G*_**	***c*_*B*_**
Angry	231	30	36
Disgust	97	45	145
Fear	246	145	31
Happy	74	184	71
Neutral	255	255	255
Sad	26	97	175
Surprise	251	233	37

Considering each RGB channel as an independent component, we can define the network's output to be *y*∈IR^7^. Then, the RGB values given by:


(10) $$\begin{array}{l} \begin{array}{c} R=y^{\text{T}}c_{R}\\ G=y^{\text{T}}c_{G}\\ B=y^{\text{T}}c_{B} \end{array} \end{array}$$

where $c_{R},c_{G},c_{B}\in {\text{IR}}^{7}$ are, respectively, the vectors of the red, green, and blue components of the pure emotions colors (Table 5).

## 6 Conclusion

In this work, we build a complete pipeline for a Facial Expression Recognition (FER) system. Our main contribution is the introduction of a rather simple attention mechanism, taking inspiration from the recent state of the art. Through a comparative test, we shown that the introduction of the proposed attention mechanism does improve marginally (~1%) the accuracy rate of the preexisting model, and also of other deeper model. With this we conclude that the model effectiveness does not depend on the employed backbone CNN. In particular, the Custom VGGFace with STN+MSA modules has achieved competitive performance (in terms of accuracy) in the experiments that have been performed, with a prediction accuracy of 82.54%.

Moreover, we employed the latter system to resonate with the users mood though dynamic environment change. In particular, the probability distribution coming from the neural model is used as a continuous signal which controls the RGB values of a LED light. This last part bases the emotion-color association on psychological studies such as Plutchik (2001), but also independent surveys (Jonauskaite et al., 2019) could be used as well like in Gupta and Gupta (2020).

The system presented in this paper has a wide field of employment. It's aim is to detect and point out a person's emotion in a very simple and informative way through colors. Applications of such systems can be useful when the environment needs to intelligently adapt to the user, i.e., changing the light color in response to people mood in a room full of music stimuli, but we also acknowledge the possibility of misuse. Indeed, emotions are a fundamental part of peoples live and thus are private. Advancing the state of the art in this field also means exposing every human inner self to the world. As for most of the research the pros and cons must be weighted and evaluated considering every possible use case scenario. We believe that our work does not hold enough practical ground to be misused by third actors, but we are still concerned with this possibility.

## Data Availability

The original contributions presented in the study are included in the article/supplementary material, further inquiries can be directed to the corresponding author.

## Footnotes

^1^*Neutral, fear, surprise, anger, disgust, happiness, sadness*.

^2^Some datasets may include more, e.g., with the addition of the *neutral* emotion class.

^3^Even though it does so by training on a set of designer-specified ground truth binary masks that try to encode the idea of varying granularity.

^4^Inputs are cropped from the center and four corners of the image and then flipped horizontally.

^5^Link available at https://github.com/omarsayed7/Deep-Emotion.

^6^The true positive rate is approximately 6% greater.

## References

[B1] Ali M. Rabehi A. Souahlia A. Guermoui M. Teta A. Tibermacine I. E.. (2025). Enhancing pv power forecasting through feature selection and artificial neural networks: a case study. Sci. Rep. 15:22574. 10.1038/s41598-025-07038-x 40595136 PMC12215858

[B2] Assari M. A. Rahmati M. (2011). “Driver drowsiness detection using face expression recognition,” in 2011 IEEE International Conference on Signal and Image Processing Applications (ICSIPA) (Kuala Lumpur: IEEE), 337–341.

[B3] Asthana A. Zafeiriou S. Cheng S. Pantic M. (2014). “Incremental face alignment in the wild,” in Proceedings of the IEEE Conference on Computer Vision and Pattern Recognition (Columbus, OH: IEEE), 1859–1866.

[B4] Barsoum E. Zhang C. Canton Ferrer C. Zhang Z. (2016). “Training deep networks for facial expression recognition with crowd-sourced label distribution,” in ACM International Conference on Multimodal Interaction (ICMI) (New York: ACM).

[B5] Bouchelaghem S. Tibermacine I. E. Balsi M. Moroni M. Napoli C. (2024). “Cross-domain machine learning approaches using hyperspectral imaging for plastics litter detection,” in 2024 IEEE Mediterranean and Middle-East Geoscience and Remote Sensing Symposium (M2GARSS) (Oran: IEEE), 36–40.

[B6] Boutarfaia N. Russo S. Tibermacine A. Tibermacine I. E. (2023). Deep learning for eeg-based motor imagery classification: Towards enhanced human-machine interaction and assistive robotics. Life 2:4.

[B7] Ca namero L. (2003). Designing emotions for activity selection in autonomous agents. Emot. Humans Artif. 115:148. 10.7551/mitpress/2705.003.000530936572

[B8] Darwin C. Ekman P. Prodger P. (2002). The Expression of the Emotions in Man and Animals. Oxford: Oxford University Press.

[B9] Dhall A. Goecke R. Lucey S. Gedeon T. (2012). Collecting large, richly annotated facial-expression databases from movies. IEEE multimedia 19:34–41. 10.1109/MMUL.2012.26

[B10] eddine Boukredine S. Mehallel E. Boualleg A. Baitiche O. Rabehi A. Guermoui M.. (2025). Enhanced performance of microstrip antenna arrays through concave modifications and cut-corner techniques. ITEGAM-JETIA 11, 65–71. 10.5935/jetia.v11i51.1414

[B11] Ekman P. (1992). An argument for basic emotions. Cognit. Emot. 6, 169–200. 10.1080/02699939208411068

[B12] Ekman P. Friesen W. V. (1971). Constants across cultures in the face and emotion. J. Pers. Soc. Psychol. 17:124. 10.1037/h0030377 5542557

[B13] Faraj Z. Selamet M. Morales C. Torres P. Hossain M. Chen B.. (2021). Facially expressive humanoid robotic face. HardwareX 9:e00117. 10.1016/j.ohx.2020.e00117 35492039 PMC9041256

[B14] Farzaneh A. H. Qi X. (2021). “Facial expression recognition in the wild via deep attentive center loss,” in Proceedings of the IEEE/CVF Winter Conference on Applications of Computer Vision (Waikoloa, HI: IEEE), 2402–2411.

[B15] Gan Y. Chen J. Yang Z. Xu L. (2020). Multiple attention network for facial expression recognition. IEEE Access 8, 7383–7393. 10.1109/ACCESS.2020.2963913

[B16] Goodfellow I. J. Erhan D. Carrier P. L. Courville A. Mirza M. Hamner B.. (2013). “Challenges in representation learning: a report on three machine learning contests,” in International Conference on Neural Information Processing (Cham: Springer), 117–124. 10.1016/j.neunet.2014.09.005 25613956

[B17] Gupta S. Gupta S. K. (2020). Investigating emotion-color association in deep neural networks. arXiv:2011.11058. 10.48550/arXiv.2011.11058

[B18] Gursesli M. C. Lombardi S. Duradoni M. Bocchi L. Guazzini A. Lanata A. (2024). Facial emotion recognition (FER) through custom lightweight cnn model: performance evaluation in public datasets. IEEE Access 12, 45543–45559. 10.1109/ACCESS.2024.3380847

[B19] Hamm J. Kohler C. G. Gur R. C. Verma R. (2011). Automated facial action coding system for dynamic analysis of facial expressions in neuropsychiatric disorders. J. Neurosci. Methods 200, 237–256. 10.1016/j.jneumeth.2011.06.023 21741407 PMC3402717

[B20] Hanson D. Pioggia G. Dinelli S. Di Francesco F. Francesconi R. de Rossi D. (2002). Identity Emulation (IE): Bio-Inspired Facial Expression Interfaces for Emotive Robots (Proceedings of AAAI Conference), 72–82.

[B21] Ioffe S. Szegedy C. (2015). “Batch normalization: accelerating deep network training by reducing internal covariate shift,” in International conference on machine learning, pages 448-456. PMLR.35496726

[B22] Jaderberg M. Simonyan K. Zisserman A. Kavukcuoglu K. (2015). Spatial transformer networks. Adv. Neural Inf. Process. Syst. 28, 2017–2025.

[B23] Jonauskaite D. Wicker J. Mohr C. Dael N. Havelka J. Papadatou-Pastou M.. (2019). A machine learning approach to quantify the specificity of colour-emotion associations and their cultural differences. R. Soc. Open Sci. 6:190741. 10.1098/rsos.190741 31598303 PMC6774957

[B24] Kaulard K. Cunningham D. W. Bülthoff H. H. Wallraven C. (2012). The mpi facial expression database—a validated database of emotional and conversational facial expressions. PLoS ONE 7:e32321. 10.1371/journal.pone.0032321 22438875 PMC3305299

[B25] Kingma D. P. Ba J. (2014). Adam: A method for stochastic optimization. arXiv [preprint] *arXiv*:1412.6980. 10.48550/arXiv.1412.6980

[B26] Ko B. C. (2018). A brief review of facial emotion recognition based on visual information. Sensors 18:401. 10.3390/s18020401 29385749 PMC5856145

[B27] Lee M.-F. Chen G.-S. (2013). “Backpropagation neural network model for detecting artificial emotions with color,” in 2013 International Joint Conference on Awareness Science and Technology Ubi-Media Computing (iCAST 2013 UMEDIA 2013) (Aizu-Wakamatsu: IEEE), 433–438. 10.1109/ICAwST.2013.6765479

[B28] Li S. Deng W. (2020). Deep facial expression recognition: a survey. IEEE Trans. Affect. Comp. 13, 1195–1215. 10.1109/TAFFC.2020.2981446

[B29] Li S. Deng W. Du J. (2017). “Reliable crowdsourcing and deep locality-preserving learning for expression recognition in the wild,” in Proceedings of the IEEE Conference on Computer Vision and Pattern Recognition (Honolulu, HI: IEEE), 2852–2861.

[B30] Li X. Zhu C. Yang S. (2025). Facial expression recognition in the wild: A new adaptive attention-modulated contextual spatial information network. Comp. Elect. Eng. 124:110258. 10.1016/j.compeleceng.2025.11025835885106

[B31] Lucey P. Cohn J. F. Kanade T. Saragih J. Ambadar Z. Matthews I. (2010). “The extended cohn-kanade dataset (ck+): A complete dataset for action unit and emotion-specified expression,” in 2010 IEEE Computer Society Conference on Computer Vision and Pattern Recognition-Workshops (San Francisco, CA: IEEE), 94–101.

[B32] Lyons M. Akamatsu S. Kamachi M. Gyoba J. (1998a). “Coding facial expressions with gabor wavelets,” in Proceedings Third IEEE International Conference on Automatic Face and Gesture Recognition (Nara: IEEE), 200–205.

[B33] Lyons M. Kamachi M. Gyoba J. (1998b). The Japanese Female Facial Expression (Jaffe) Dataset.

[B34] Ma F. Sun B. Li S. (2022). Spatio-temporal transformer for dynamic facial expression recognition in the wild. arXiv [preprint] arXiv:2205.04749. 10.48550/arXiv.2205.04749

[B35] Maalej A. Amor B. B. Daoudi M. Srivastava A. Berretti S. (2011). Shape analysis of local facial patches for 3D facial expression recognition. Pattern Recognit. 44, 1581–1589. 10.1016/j.patcog.2011.02.012

[B36] Martınez-Miranda J. Aldea A. (2005). Emotions in human and artificial intelligence. Comput. Human Behav. 21, 323–341. 10.1016/j.chb.2004.02.010

[B37] Mehrabian A. (2017). “Communication without words,” in Communication Theory (London: Routledge), 193–200.

[B38] Meng D. Peng X. Wang K. Qiao Y. (2019). “Frame attention networks for facial expression recognition in videos,” in 2019 IEEE International Conference on Image Processing (ICIP) (Taipei: IEEE), 3866–3870.

[B39] Minaee S. Minaei M. Abdolrashidi A. (2021). Deep-emotion: Facial expression recognition using attentional convolutional network. Sensors 21:3046. 10.3390/s21093046 33925371 PMC8123912

[B40] Mollahosseini A. Hasani B. Mahoor M. H. (2017). Affectnet: a database for facial expression, valence, and arousal computing in the wild. IEEE Trans. Affect. Comp. 10, 18–31. 10.1109/TAFFC.2017.274092332380751

[B41] Naidji I. Tibermacine A. Guettala W. Tibermacine I. E. (2023). “Semi-mind controlled robots based on reinforcement learning for indoor application,” in ICYRIME (CEUR), 51–59.

[B42] Naz K. Helen H. (2004). “Color-emotion associations: Past experience and personal preference,” in AIC 2004 Color and Paints, Interim Meeting of the International Color Association.

[B43] Pantic M. Valstar M. Rademaker R. Maat L. (2005). “Web-based database for facial expression analysis,” in 2005 IEEE International Conference on Multimedia and Expo (Amsterdam: IEEE).

[B44] Paszke A. Gross S. Massa F. Lerer A. Bradbury J. Chanan G.. (2019). “Pytorch: an imperative style, high-performance deep learning library,” in Advances in Neural Information Processing Systems 32, eds. H. Wallach, H. Larochelle, A. Beygelzimer, F. d' Alché-Buc, E. Fox, and R. Garnett (New York: Curran Associates, Inc), 8024–8035.

[B45] Plutchik R. (2001). The nature of emotions. Am. Sci. 89:344. 10.1511/2001.28.344

[B46] Ram V. Schaposnik L. P. Konstantinou N. Volkan E. Papadatou-Pastou M. Manav B.. (2020). Extrapolating continuous color emotions through deep learning. Phys. Rev. Res. 2:3. 10.1103/PhysRevResearch.2.033350

[B47] Ren S. Cao X. Wei Y. Sun J. (2014). “Face alignment at 3000 fps via regressing local binary features,” in Proceedings of the IEEE Conference on Computer Vision and Pattern Recognition (Columbus, OH: IEEE), 1685–1692.

[B48] Rogers M. A. Zaragoza-Lao E. (2003). Happiness and children's health: an investigation of art, entertainment, and recreation. Am. J. Public Health 93:288–289. 10.2105/AJPH.93.2.288 12554587 PMC1447731

[B49] Russo S. Ahmed S. Tibermacine I. E. Napoli C. (2024a). “Enhancing eeg signal reconstruction in cross-domain adaptation using cyclegan,” in 2024 International Conference on Telecommunications and Intelligent Systems (ICTIS) (Djelfa: IEEE), 1–8.

[B50] Russo S. Tibermacine I. E. Tibermacine A. Chebana D. Nahili A. Starczewscki J.. (2024b). Analyzing EEG patterns in young adults exposed to different acrophobia levels: a VR study. Front. Hum. Neurosci. 18:1348154. 10.3389/fnhum.2024.1348154 38770396 PMC11102978

[B51] Shen P. Wang S. Liu Z. (2013). Facial expression recognition from infrared thermal videos. Intellig. Autonom. Syst. 12, 323–333. 10.1007/978-3-642-33932-5_31

[B52] Shorten C. Khoshgoftaar T. M. (2019). A survey on image data augmentation for deep learning. J. Big Data 6:1–48. 10.1186/s40537-019-0197-0 PMC8287113 34306963

[B53] Simonyan K. Zisserman A. (2014). Very deep convolutional networks for large-scale image recognition. arXiv [preprint] arXiv:1409.1556. 10.48550/arXiv.1409.1556

[B54] Srivastava N. Hinton G. Krizhevsky A. Sutskever I. Salakhutdinov R. (2014). Dropout: a simple way to prevent neural networks from overfitting. J. Mach. Learn. Res. 15, 1929–1958.33259321

[B55] Sujono and Gunawan A. A.. (2015). Face expression detection on kinect using active appearance model and fuzzy logic. Procedia Comput. Sci. 59, 268–274. 10.1016/j.procs.2015.07.558

[B56] Sutskever I. Martens J. Dahl G. Hinton G. (2013). “On the importance of initialization and momentum in deep learning,” in International Conference on Machine Learning (New York: PMLR), 1139–1147.

[B57] Tibermacine A. Akrour D. Khamar R. Tibermacine I. E. Rabehi A. (2024a). “Comparative analysis of SVM and CNN classifiers for EEG signal classification in response to different auditory stimuli,” in 2024 International Conference on Telecommunications and Intelligent Systems (ICTIS) (Djelfa: IEEE), 1–8.

[B58] Tibermacine A. Guettala W. Tibermacine I. E. (2024b). Efficient one-stage deep learning for text detection in scene images. Electrotehnica, Electronica, Automatica 72:1108007. 10.46904/eea.24.72.4.1108007

[B59] Tibermacine A. Tibermacine I. E. Zouai M. Rabehi A. (2024c). “EEG classification using contrastive learning and riemannian tangent space representations,” in 2024 International Conference on Telecommunications and Intelligent Systems (ICTIS) (Djelfa: IEEE), 1–7.

[B60] Tibermacine I. E. Russo S. Citeroni F. Mancini G. Rabehi A. Alharbi A. H.. (2025a). Adversarial denoising of EEG signals: a comparative analysis of standard gan and WGAN-GP approaches. Front. Hum. Neurosci. 19:1583342. 10.3389/fnhum.2025.1583342 40395688 PMC12089060

[B61] Tibermacine I. E. Tibermacine A. Guettala W. Napoli C. Russo S. (2023). “Enhancing sentiment analysis on seed-iv dataset with vision transformers: a comparative study,” in Proceedings of the 2023 11th International Conference on Information Technology: IoT and Smart City (ACM), 238–246.

[B62] Tibermacine I. E. Tibermacine A. Zouai M. Russo S. Bouchelaghem S. Napoli C. (2025b). “Enhanced EEG classification via riemannian normalizing flows and deep neural networks,” in 2025 International Symposium on iNnovative Informatics of Biskra (ISNIB), (Biskra: IEEE), 1–6.

[B63] Vaswani A. Shazeer N. Parmar N. Uszkoreit J. Jones L. Gomez A. N.. (2017). “Attention is all you need,” in Advances in Neural Information Processing Systems, 5998–6008.

[B64] Wang K. Peng X. Yang J. Meng D. Qiao Y. (2020). Region attention networks for pose and occlusion robust facial expression recognition. IEEE Trans. Image Proc. 29, 4057–4069. 10.1109/TIP.2019.2956143 32011249

[B65] Wei W. Jia Q. Chen G. (2016). “Real-time facial expression recognition for affective computing based on kinect,” in 2016 IEEE 11th Conference on Industrial Electronics and Applications (ICIEA) (Hefei: IEEE), 161–165.

[B66] Xiang J. Zhu G. (2017). “Joint face detection and facial expression recognition with mtcnn,” in 2017 4th international conference on information science and control engineering (ICISCE) (Changsha: IEEE), 424–427.

[B67] Zhan C. Li W. Ogunbona P. Safaei F. (2008). A real-time facial expression recognition system for online games. Int. J. Comp. Games Technol. 2008:542918. 10.1155/2008/542918

[B68] Zhang H. Goodfellow I. Metaxas D. Odena A. (2019). “Self-attention generative adversarial networks,” in International Conference on Machine Learning (New York: PMLR), 7354–7363.

